# Whey protein enhances normal inflammatory responses during cutaneous wound healing in diabetic rats

**DOI:** 10.1186/1476-511X-10-235

**Published:** 2011-12-14

**Authors:** Hossam Ebaid, Amir Salem, Abdalla Sayed, Ali Metwalli

**Affiliations:** 1 Department of Zoology, College of Science, King Saud University, P.O. Box 2455, Riyadh 11451, Saudi Arabia; 2 Department of Zoology, Faculty of Science, El-Minia University, El-Minia, Egypt; 3 Department of Mathematical and Life Sciences, Graduate School of Science, Hiroshima University, Higashi-Hiroshima 739-8526, Japan; 4 Department of Pathology, Medical Research Division, National Research Center, Cairo, Egypt; 5 Department of Food Science, College of Agriculture and Food Science, King Saud University, Riyadh, Saudi Arabia; 6 Department of Dairy, Faculty of Agriculture, El-Minia University, El-Minia, Egypt

**Keywords:** Whey protein, diabetes, wound healing, oxidative stress, inflammatory cytokines

## Abstract

**Background:**

Prolonged wound healing is a complication of diabetes that contributes to mortality. Impaired wound healing occurs as a consequence of excessive reactive oxygen species (ROS) production. Whey protein (WP) is able to reduce the oxygen radicals and increase the levels of the antioxidant glutathione. Thus, the aim of this study was to determine whether dietary supplementation with WP could enhance normal inflammatory responses during wound healing in diabetic rats. Animals were assigned into a wounded control group (WN), a wounded diabetic group (WD) and a wounded diabetic group orally supplemented with whey protein (WDWP) at a dose of 100 mg/kg body weight.

**Results:**

Whey protein was found to significantly decrease the levels of malondialdehyde (MDA), nitric oxide (NO) and ROS. A significant restoration of the glutathione level was observed in WDWP rats. During the early wound healing stage, IL-1β, TNF-α, IL-6, IL-4 and neutrophil infiltration were significantly decreased in WD mice. WP supplementation was found to restore the levels of these inflammatory markers to the levels observed in control animals. In addition, the time required for wound healing was significantly prolonged in diabetic rats. WP was found to significantly decrease the time required for wound healing in WDWP rats.

**Conclusion:**

In conclusion, dietary supplementation with WP enhances the normal inflammatory responses during wound healing in diabetic mice by restoring the levels of oxidative stress and inflammatory cytokines.

## Background

The ability of animals to repair wounds is critical for survival after injury [1]. A multitude of cellular events, such as cell proliferation, cell migration, contraction and extracellular matrix degradation and synthesis, must occur to achieve wound closure and regeneration of the injured dermis [2]. These events rely on the temporal expression and activation of a variety of proteins, such as growth factors, cytokines and matrix metalloproteinases [3].

Wound healing is initiated by an inflammatory phase that is followed by a proliferation phase, particularly the proliferation of fibroblasts and endothelial cells. The last phase involves the production and reorganization of the extracellular matrix, leading to repair or regeneration. The inflammatory phase leads to the recruitment of leukocytes that produce growth factors and remove debris from the wound [4-7]. The healing process requires an interaction between inflammatory cells and biochemical mediators, which is stimulated by a number of mitogens and chemotactic factors [4].

The generation of oxygen radicals is normally balanced by the presence of adequate endogenous antioxidant defenses [8]. Oxidative stress has been implicated in the pathology of diabetes mellitus [9,10], a disease marked by a prolonged inflammatory period that increases the time required for recovery. Impaired wound healing occurs as a consequence of excessive ROS production. Identification of the dietary proteins that enhance skin repair in diabetes may contribute to the development of novel therapeutic strategies. Many studies have confirmed the role of glutathione, which is increased by dietary WP, as a powerful antioxidant [11,12]. WP is able to reduce the effects of oxygen radicals and lipid peroxidation by increasing the activity of the antioxidant glutathione, thus stimulating epithelization and the proliferation of fibroblasts as well as increasing the secretion of both pro- and post-inflammatory cytokines. WP has been found to significantly suppress hydroperoxide and ROS levels in leukocytes, liver and cutaneous tissues in mice by restoring the antioxidant glutathione [13].

In addition, WP contains all of the essential and non-essential amino acids and is a good source of glutamine and the branched-chain amino acids that are necessary for cell growth [14]. The branch-chained amino acids leucine, isoleucine and valine promote the healing of bones, skin and muscle tissues. The amino acid proline aids in the production of collagen, thereby healing cartilage and strengthening joints, tendons and cardiac muscle [15].

We hypothesized that wound healing in diabetic animals could be improved by supplementing their diets with WP. Data from our previous work indicate that WP increases the capacity of non-diabetic animals to heal wounds [16]. Additionally, our recent study [13] revealed the potential effects of WP on immune processes, including the regulation of many cytokines. We also found that the ability of peripheral blood mononuclear cells (PBMCs) to proliferate in response to stimulation with different antigens was significantly increased in the WP-treated group [17].

## Results

Impaired wound healing in diabetes occurs as a consequence of excessive ROS production. WP is known to result in the reduction of the oxygen radicals and increase levels antioxidant glutathione. Thus, we aimed to accelerate the wound healing process using a dietary supplementation of whey protein in a streptozotocin-diabetic rat model. External indices of the wound size over time, oxidative stress and the inflammatory markers were analyzed in this study to evaluate the effect of whey protein on this vital process.

### Whey protein enhanced wound closure rate in diabetic models

The time required to heal wounds in diabetic rats was prolonged. Interestingly, results showed that the time required to heal wounds was significantly shortened in WDWP rats comparing to the diabetic rats. All wounded diabetic animals fed whey protein achieved complete healing by day 8. Within 8 days of wounding, 100% of the WDWP and WN rats' wounds showed complete closure, whereas only 20% of WD wounds exhibited complete closure by D12 (Table 1). In addition, 30% of the diabetic rats did not survive past day 10.

**Table 1:** Percentage of animals that achieved complete wound healing by day 6 (D6), day 8 (D8) and day 10 (D10) in non-diabetic normal rats (WN), diabetic rats (WD) and diabetic rats supplemented with whey protein (WDWP) and the mortality rate in these groups by D10

	Complete Healing %			Mortality at D10 %
	
	D6	D8	D10	
**WN**	40%	100%	100%	0%
**WD**	0%	0%	20%	30%
**WDWP**	40%	100%	100%	0%

**Figure 1:**
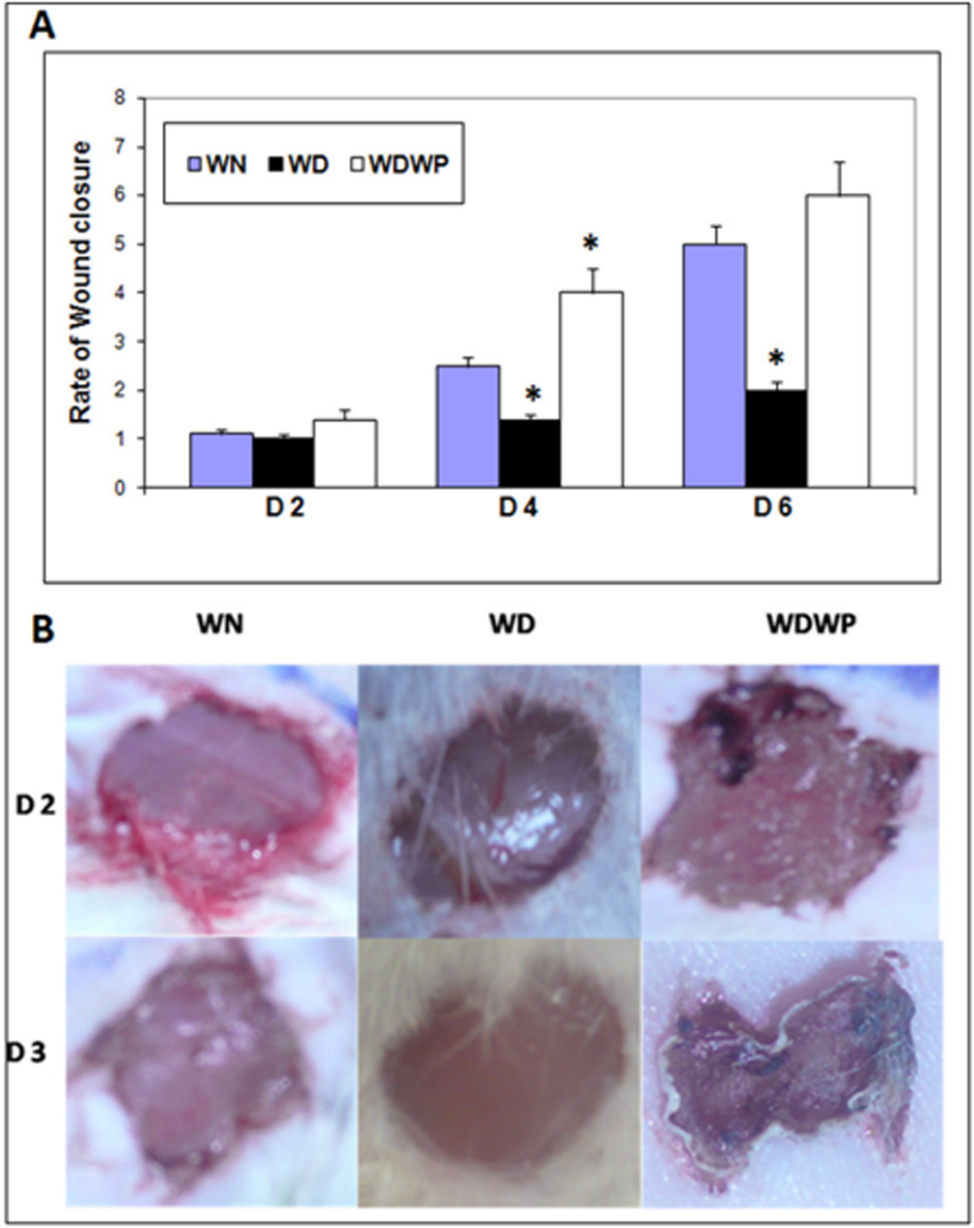
**Wound closure rate in non-diabetic normal rats (WN), diabetic rats (WD) and diabetic rats supplemented with whey protein (WDWP)**. A: Wound closure rate at two days intervals showing the effect of whey protein on wound healing in WDWP rats that recorded a significantly faster closure rate than that of the diabetic rats. B: External photograph showing wounds in the different groups at D2 and D3. Wound contraction is obvious in the WDWP animals at D3. No visible changes had occurred in the diabetic wounds at the D3. Wound size was calculated by determining the area of the wound each day in comparison to the area of the standard square.

On day 2, no significant differences were observed among different rat groups. However, on day 3, it was clear that the wound closure rate in the WD animals was significantly delayed due to the effect of diabetes on the inflammatory stage of healing. WP was found to significantly (*P *< 0.05) accelerate the closure rate in diabetic rats (WDWP) by 2.5 fold more than that of the diabetic rats (WD) on days 3 and 6, and the closure rate in WDWP rats was also significantly higher than the control rats on day 3 (Figure 1A).

In addition, wound contraction was obvious in the wounds of the WDWP group at day 3 and in the wounds of control rats at day 5. However, no wound contraction was observed in the diabetic wound group until day 10, and the contraction was poor in two rats. In summary, the rate of wound closure in the whey protein supplemented diabetic group was significantly faster than those of the diabetic group **(ex. on **Figure 1B).

### Whey protein improved parameters of oxidative stress

**Figure 2:**
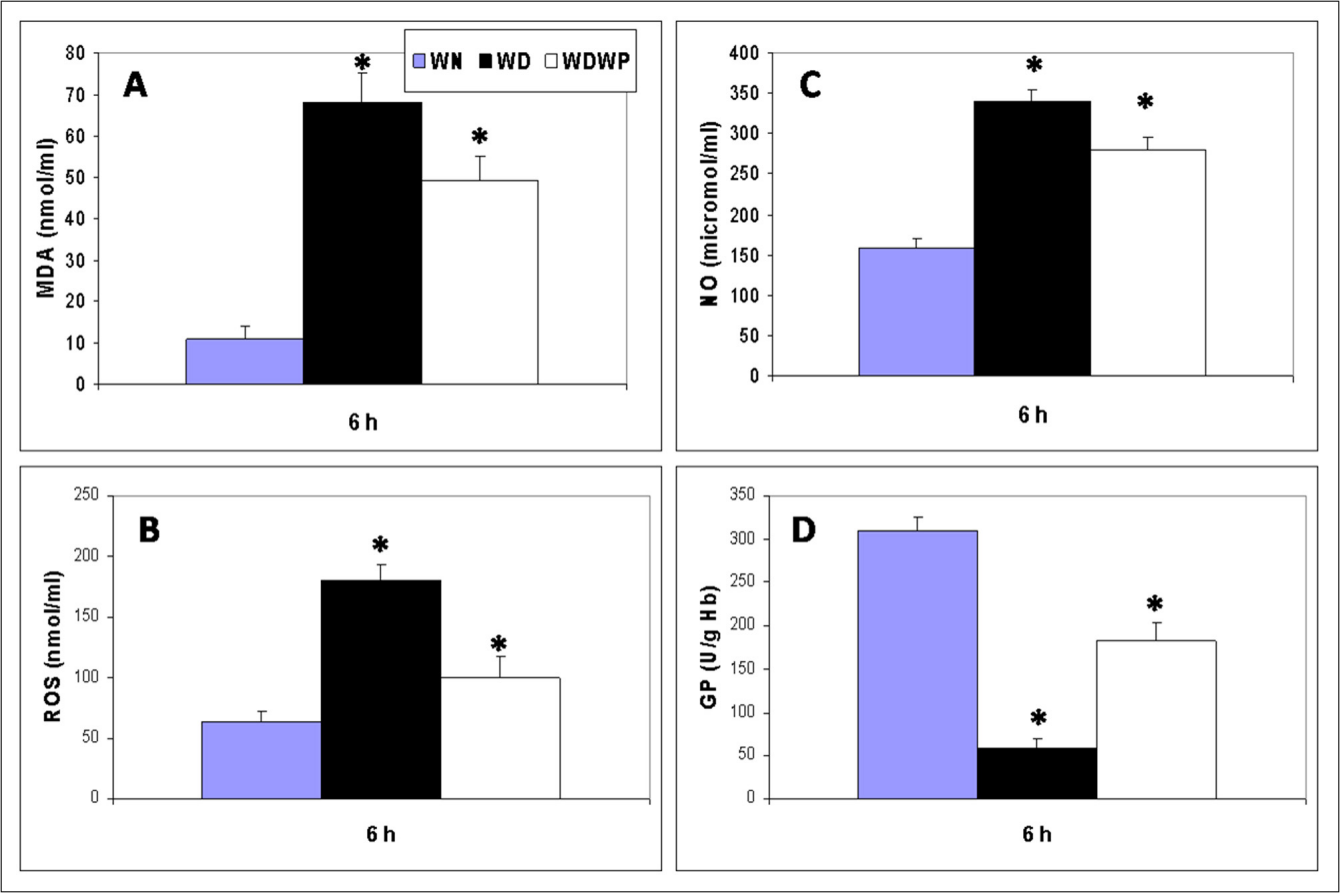
**Oxidative stress in non-diabetic normal rats (WN), diabetic rats (WD) and diabetic rats supplemented whey protein (WDWP)**. MDA, NO and total glutathione were measured in liver tissues, while ROS was estimated in skin tissue. All oxidative markers were significantly elevated in the diabetic rats. Whey protein restored the levels of these markers in WDWP rats to levels close to those of the control rats and significantly increased glutathione levels.

Because oxidative stability is vital for normal wound healing, we monitored the oxidative stress in WN, WD and WDWP rats. As expected, all oxidative markers were significantly elevated in the diabetic rats. A significant inhibition (*P *< 0.05) of the oxidative parameters was observed in the WDWP rats. Statistical analysis revealed that the rat group supplemented with WP showed a significant decrease in the level of MDA compared to the control mice. We also observed a significant suppression of both NO and ROS in the WDWP group. Interestingly, glutathione was significantly restored (*P *< 0.05) in the WDWP rats (Figure 2), nearly reaching the level observed in control animals.

### Whey protein stimulated inflammatory cytokines

**Figure 3:**
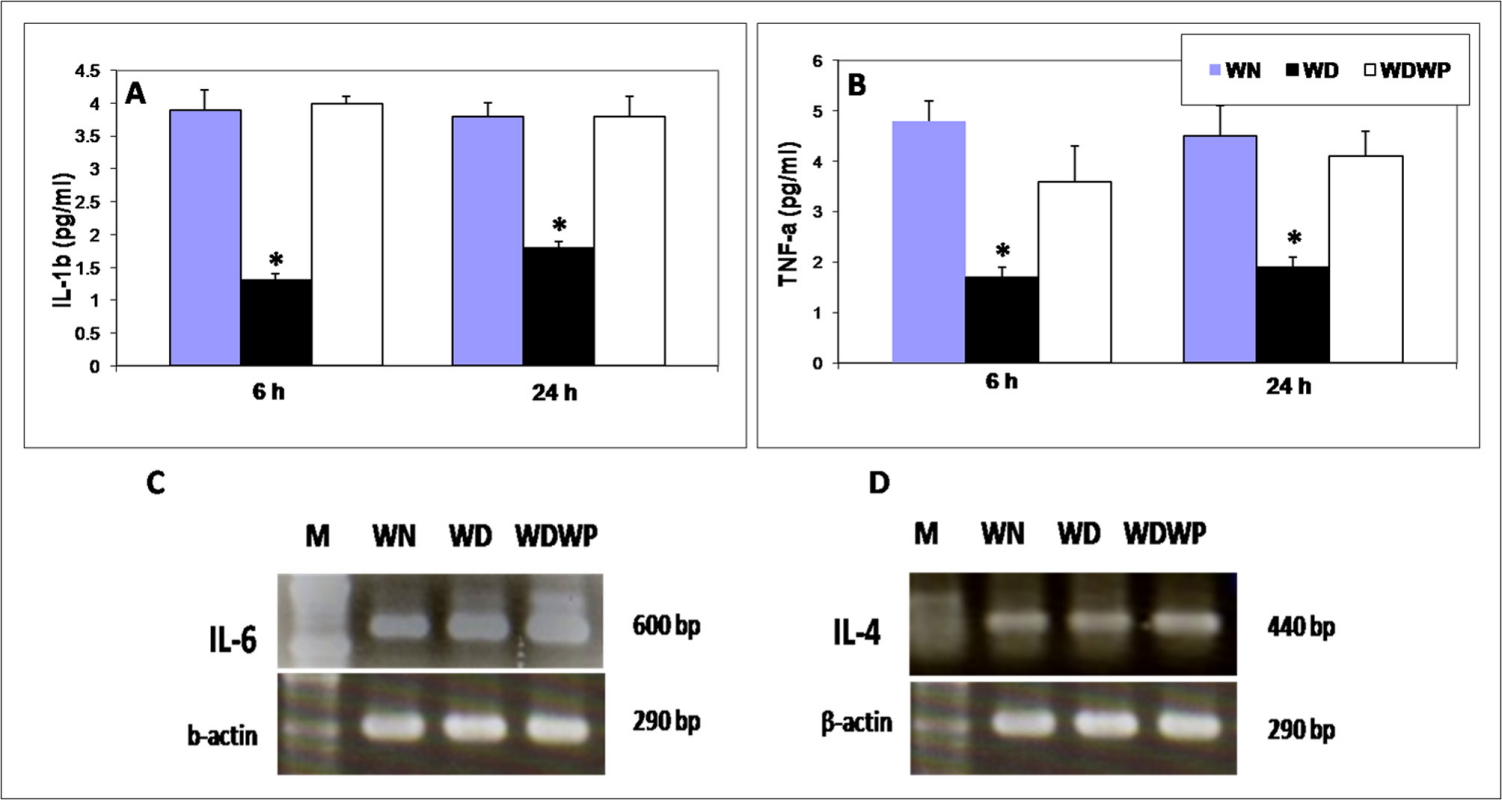
**A, B: ELISA estimation of both IL-1β and TNF-α levels at 6 h and 24 h in wounded non-diabetic normal rats (WN), wounded diabetic rats (WD) and wounded diabetic rats supplemented with whey protein (WDWP)**. C,D: Gene expression of the IL-6 (6 h) and IL-4 (24 h) proteins in the three groups showing a notable effect of the whey protein on both cytokines after wounding.

Activated tissue macrophages secrete three cytokines (IL-1β, TNF-α and IL-6) that induce many of the localized and systemic changes observed in the acute phase of the inflammatory response. Thus, the pro-inflammatory cytokines IL-1β and TNF-α were measured to determine their roles in the inflammatory stage of the wound healing process. The level of IL-1β peaked at 6 h in both the control WN rats and the WDWP rats (Figure 3A), but low levels of IL-1β were observed in the WD rats until 24 h. Moreover, the level of IL-1b in the WD group was significantly lower than the levels of IL-1β in either the control or WDWP rats. Notably, the pattern of TNF-α levels was similar to that of IL-1β in all groups (Figure 3B). The level of TNF-α was significantly lower in WD rats compared to control or WDWP rats.

At the beginning of the inflammatory stage, IL-6 is secreted by macrophages locally and systemically and induces many changes, including changes in vascular permeability [18]. Therefore, we examined IL-6 levels 6 h after wounding. The IL-6 level was significantly elevated in WP supplemented rats (WDWP) compared to the diabetic group (WD) (Figure 3C).

Macrophage-derived cytokines, such as IL-4, are responsible for tissue formation. Therefore, we detected IL-4 24 h after wounding. The results showed that IL-4 levels were decreased in the diabetic rats. We found that whey protein significantly elevated the IL-4 levels in the diabetic rats (WDWP), which were approximately twice the level of the diabetic animals (WD), as shown in Figure 3D.

### Whey protein stimulated neutrophil infiltration into wound sites

**Figure 4:**
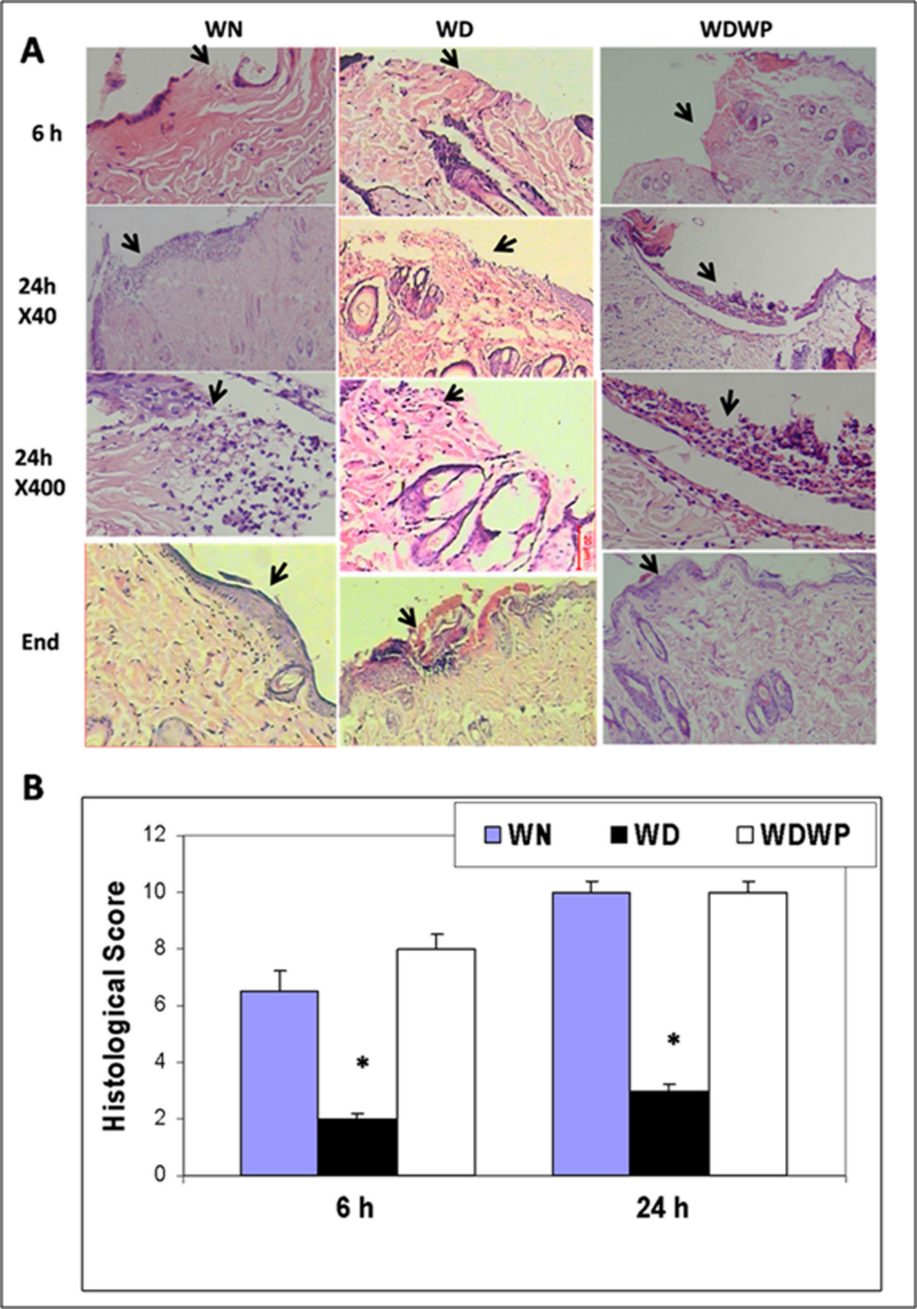
**A: Representative photographs from the vertical sections of the wound sites from different groups after 6 h (H&E ×40), 24 h (H&E ×40, second row; H&E ×400, third row) and at the end of the experiment (H&E ×40)**. The second row of these sections shows higher magnifications of the wound sites infiltrated with large number of inflammatory cells, especially neutrophils, in both WN and WDWP rats. A small number of the inflammatory cells are shown in the section from WD rats (arrow head). B: Neutrophil infiltration in wound sites in non-diabetic normal rats (WN), diabetic rats (WD) and diabetic rats supplemented whey protein (WDWP). A score between 0 (no infiltration) and 10 (neutrophil infiltration in most areas) for neutrophil infiltrations was given for each investigated section.

The histological sections of wounded skin revealed that the epidermal cell proliferation and migration, and the dermal reorganization gradually improved and was complete by day 8 in WDWP animals. However, the phases of wound healing were delayed and disturbed in WD counterparts and the epidermal cell migration only partially covered the wound region. Additionally, hair follicles, which reflect dermal contraction, were rarely observed in the dermis (Figure 4A).

In the early stages of an inflammatory response, the predominant cell type that infiltrates the tissue is neutrophils. Neutrophil infiltration peaks within the first 6 hours [18]. Neutrophil infiltration was significantly depressed in diabetic rats compared to control and WDWP rats at 6 and 24 h after wounding. Thus, diabetes leads to a quantitative reduction in the level of neutrophil infiltration into the wound site up to 24 h after wounding. On the other hand, the level of neutrophil infiltration was restored to control levels in the WDWP rats (Figure 4B). Taken together, these findings demonstrate that the early inflammatory response is dramatically attenuated in WD rats and that supplementation of WDWP rats with WP effectively restores the features of the early inflammatory response to levels observed in control mice.

## Discussion

Accelerating wound healing in diabetes is the aim of a large number of studies. As a result of excessive ROS, a disturbed early inflammatory phase of healing characterizes diabetic wounds [19]. In this study, we have demonstrated that wound closure is significantly shortened in diabetic rats supplemented with WP in a cutaneous model of wound healing. Moreover, our data clearly demonstrated that WP supplementation of diabetic animals reduced oxidative stress and restored markers of the inflammatory response to levels similar to control animals.

Oxidative stress occurs due to an imbalance between the production of ROS and the protection by cellular antioxidants [20]. During the inflammatory phase, the release of oxygen radicals by neutrophils and macrophages causes tissue damage [21]. We found that the levels of MDA, NO and ROS were elevated in diabetic rats and that WP restored the oxidative markers in WDWP rats. Previous studies have shown that WP plays an active role in iron transport [22], in the cytotoxic defense of neutrophils [23], and in scavenging free radicals [24]. Indeed, a significant enhancement of glutathione was observed in the WP supplemented rats in this study. WP has been shown to be a potent inducer of glutathione. The ability of cysteine to increase glutathione levels is greater when it is delivered in whey protein rather than as free cysteine [25].

Therefore, WP is able to reduce the effects of oxygen radicals and lipid peroxidation by increasing the antioxidant glutathione and thus stimulating the early normal inflammatory events of the healing process. Accordingly, WP has a potential role in stimulating the characteristic inflammatory cytokines. WP restored high levels of both IL-1β, TNF-α in diabetic rats supplemented with WP (WDWP). IL-1β, TNF-α modulate the expression of the chemokines and adhesion molecules necessary for the recruitment of inflammatory cells to the site of injury [26,27]. A significant increase in neutrophil infiltration into the wound sites was observed in WDWP rats and control rats compared in the diabetic rats. These findings further suggest that WP inhibits ROS and other oxidative markers and elevates neutrophil infiltration during the early inflammatory response in WDWP rats. We previously found that the *in vitro *chemotaxis of B cells, T cells and bone marrow-derived dendritic cells toward CCL-21 and CXCL-12 was significantly increased in WP-treated mice [17].

Neutrophils are major producers of numerous cytokines that serve as some of the earliest signals to activate keratinocytes and fibroblasts, thus initiating the proliferative and remodeling phases of wound healing [28,29]. Neutrophils are the first inflammatory cells to infiltrate the wound site and play a key role during the early inflammatory stage of wound healing by clearing bacteria and phagocytosing cellular debris [30]. It was found that bovine whey protein primes normal human blood neutrophils by enhancing chemotaxis, phagocytosis, oxidative burst, and degranulation [31].

Pradhan et al. [32] found that the post-injury expression of IL-6, IL-8, CXCR1, CXCR2, and GP-130 is significantly less in diabetic wounds compared with non-diabetic wounds. WP was found to stimulate the accumulation of IL-1β, IL-8, IL-6, macrophage inflammatory protein (MIP)-1α, MIP-1β and TNF-α [31]. Similarly, we found that WP stimulated higher levels of IL-6 in WDWP rats than diabetic rats.

In diabetic rats, lipid peroxidation of cellular and organelle membranes, disruption of the intracellular matrix, and alteration of important protein enzymatic processes causes tissue damage [21]. Tissue damage impairs growth and proliferation of keratinocyte endothelial cells, fibroblasts, and collagen metabolism [33,34], which in turn can impair the healing process (delayed granulation tissue formation, decreased collagen, and tissue organization) [35]. We found poor re-epithelization and dermal construction in the skin sections of WD rats. On the other hand, stimulating the early normal inflammatory events of the healing process with WP greatly induced the normal re-epithelization and proliferation of fibroblasts. This result was confirmed by the improvement in the histological architecture of wounded skin in WDWP rats compared to WD rats (Figure 4A). Furthermore, WP significantly elevated the level IL-4 in WDWP in comparison to WD rats. IL-4 is a macrophage-derived cytokine that is responsible for tissue formation (collagen production in fibroblasts) [36]. Therefore, there is a positive correlation between the elevation of IL-4 and the normal reconstruction of the dermis in WDWP rats.

Contraction is characterized by the centripetal movement of the wound edge toward the center of the wound. Wound contraction begins 4 to 5 days after injury [37]. In our study, wound contraction was evident in the wounds of WDWP at D3 and in the wounds of control rats at D5 compared to D10 in the diabetic rats. Both myofibroblasts and fibroblasts are thought to be responsible for this process [38,39]. WP contains all amino acids, including glutamine and the branched-chain amino acids that are necessary for cell growth [14]. Therefore, in addition to a supply of glutathione and the appropriate essential amino acids, whey protein enhanced the latter stages of wound healing, such as tissue repair and remodeling, in diabetic rats. These findings clearly explained the high wound closure rate and the normal epithelization of wound healing in the WDWP rats.

Taken together, the results of this work support the hypothesis that the oxidative stability induced in diabetic rats by WP contributes to the accelerated wound healing and immune stimulation observed in WDWP rats. NF_k_B is required for the induction of pro-inflammatory cytokines, such as IL-1β, TNF-α and IL-6 [40]. Although we did not address this in the present study, oxidative stability induced by WP may mediate the activation of NF_k_B, leading to the activation of the inflammatory cascade and the stimulation of wound healing, resulting in the faster wound closure rate in WDWP rats.

Our findings explain the significant restoration of inflammatory markers in whey protein-treated diabetic rats. The current study demonstrated that WP induces the early inflammatory response of cutaneous wound healing by modulating the inflammatory response and oxidative stress. There are currently no data on the use of WP to treat wound healing impairment in diabetes. This study may provide critical insight into future nutritional intervention strategies designed to enhance early wound healing in diabetic people. This strategy would show whether this WP has a significant clinical impact. Therefore, the potential role of whey protein in the treatment of diabetic wounds needs to be intensively investigated in future research focusing on patient-oriented outcomes.

## Materials and methods

### Extraction of whey protein and animal diets

Camel milk was obtained from three breeds (Majaheem, Maghateer and sofr) of camel from the Najd region in Saudi Arabia. The milk was skimmed by centrifugation at 5000 g for 20 min using a IEC Model K centrifuge, [Boston, USA]. Skim milk was acidified to pH 4.3 using 1 M HCl. The precipitated casein was removed by centrifugation, and the supernatant containing the whey protein was saturated with ammonium sulfate (70% saturation) and incubated overnight at 4°C. The precipitated whey protein was collected by centrifugation and dialyzed against distilled water for 48 h at 4°C using a Spectra/Pro^® ^Membrane, MWCO 6000-8000 Da. The obtained dialyzates were lyophilized using a Unitop 600SL, Virtis Company, Gardiner, New York 12525 USA and were kept at -20°C until use. The dialysate containing undenatured whey proteins was freeze-dried and refrigerated until use.

### Experimental design

Adult male rats weighing 120-150 g were obtained from the College of Pharmacy, King Saud University, Saudi Arabia and housed in stainless steel wire cages (5 animals/cage) under pathogen-free conditions. All animal procedures were conducted in accordance with the standards set forth in the guidelines for the care and use of experimental animals by the Committee for the Purpose of Control and Supervision of Experiments on Animals (CPCSEA) and the National Institutes of Health (NIH) protocol. The study protocol was approved by the Animal Ethics Committee of the Zoology Department, College of Science, King Saud University. The animals were maintained at 18-22°C on a 12:12 h light/dark cycle and provided with food and water *ad libitum*.

The animals were assigned to three groups: 1) the first group was remained as a wounded non-diabetic (WN) control group and was given phosphate buffered saline, 2) the second group was a wounded diabetic group (WD) given phosphate buffered saline, 3) the third group was a wounded diabetic group orally supplemented with whey protein (WDWP) at a dose of 100 mg/kg of body weight for 15 days pre-wounding and 15 days post-wounding. Seven animals from each group were sacrificed under mild diethyl ether anesthesia 6 h, 24 h after wounding and at the end of the experiment.

### Diabetic and wound models

The diabetic groups were intraperitoneally injected with streptozotocin (65 mg/kg) to induce diabetes [41]. Streptozotocin-injected animals exhibited massive glucosuria and hyperglycemia within 5 days of injection. Diabetes was confirmed in rats by measuring the fasting blood glucose level (200-250 mg/dl) before use in this experiment.

Rats were anaesthetized with isoflurane, and the back of the rat was shaved and sterilized using an alcohol swab. The wound biopsy model used in this experiment was described previously [42] with little modification. The shaved skin was pinched and folded, and the wound was punched through the full thickness of the folded skin to form a 2 × 5 mm rectangle below the shoulder blades of each rat.

### Measurement of wound closure

The procedure for measuring wound closure was previously described by Lim et al. [19]. Wounds from individual rats were digitally photographed every day. A standard rectangle equivalent in size to the initial wound area was drawn beside the wound and used as a reference. Wound size was calculated by determining the area of the wound each day in comparison to the area of the standard rectangle. Wound closure was expressed as the ratio of the initial wound size to the wound area (each day after wounding). A higher ratio indicates faster wound closure.

### Collection of blood and tissue samples

Two blood samples were immediately collected. The first sample was used for serum analysis. Plasma was isolated from the second sample using EDTA (ethylenediaminetetra acetic acid) as an anticoagulant. Samples of plasma and serum were separated for analysis by centrifuging the blood for 15 minutes at 3000 rpm.

### Estimation of glutathione

Glutathione (GSH) assay was carried out on tissue as previously described [43]. Liver was removed and gently rinsed in physiological saline. The fresh organ weights were recorded, and organs were frozen until use. A 10% (w/v) homogenate of each frozen tissue was prepared, and the supernatant was used in the enzymatic assays. Glutathione concentrations were measured by adding 100 μl of supernatant to 400 μl PBS [containing 200 mM monochlorobenzene (MCB) and 2 U/ml glutathione S-transferase (per 100 μl)]. Glutathione concentrations were then determined by measuring the absorbance of the reaction after 1 min at 340 nm using an UV Visible Spectrometer (Ultrospec 2000, Pharmacia Biotech). Glutathione standards were measured concurrently to obtain a standard curve that was used to calculate GSH concentrations in samples. Results were expressed as μg GSH/g tissue. Statistical comparisons of GSH activities between controls and treatments in each case were performed using Minitab statistical program as detailed below.

### Estimation of nitric oxide

Nitric Oxide was assayed as described by Oliveira et al. [44]. Equal volumes of sulfanilamide (1.5% in 5% H_3_PO4) and naphthylethylene diamine dihydrochloride (0.1% in H_2_O) were mixed. An equal volume of working reagent was mixed with 0.1 ml of each liver supernatant and incubated for 15 min at room temperature. The absorbance was measured at 540 nm. The nitrite concentration was calculated by means of a NaNO_3 _standard curve, and data were expressed as millimolar nitrite.

### Estimation of lipid peroxidation

The peroxidation of the endogenous lipids in the liver homogenates was estimated spectrophotometrically following the method described by Okhawa et al., [45] and expressed in nanomoles of malondialdehyde (MDA) per milliliter of homogenate (nmole/ml). A 0.5 ml aliquot of the resulting supernatant was shaken with 2.5 ml of 20% trichloroacetic acid (TCA). To the resulting mixture, 1 ml of 0.67% thiobarbituric acid (TBA) was added, and the samples were shaken and incubated for 30 min in a boiling water bath followed by immediate rapid cooling in ice for 5 min. After cooling, 4 ml of n-butyl-alcohol was added, and the samples were shaken well. The resulting mixture was then centrifuged at 16,000 g for 5 min. The resultant n-butyl-alcohol layer was moved into a separate tube, and MDA content was determined spectrophotometrically at 535 nm using an UV Visible Spectrometer (Ultrospec 2000, Pharmacia Biotech).

### ROS measurement

ROS levels were determined using 2,7-dichlorodihydrofluorescein diacetate (H2DCF-DA) (Beyotime Institute of Biotechnology, Haimen, China). The supernatant from skin homogenates were directly treated with 10 μM H2DCF-DA dissolved in 1 ml PBS at 37°C for 20 min. The fluorescence intensity was monitored using an excitation wavelength of 488 nm and an emission wavelength of 530 nm.

### ELISA assay for the inflammatory cytokines IL-1β and TNF-α

Sera were tested for IL-1β and TNF-α by ELISA according to the manufacturer's instructions for the corresponding rat immunoassay kits (BioSource International Inc., USA). The optical densities (OD) were measured at 405 nm. The detection limits were set according to the log-log correlative coefficient of the standard curve.

### PCR determination of IL-4 and IL-6 levels

Total RNA was isolated from tissues used in this study, and mRNA was purified using Fermentas kit. The cDNA was reverse transcribed using an Oligo-dT cDNA extraction kit (Promega). cDNA was prepared using a PCR-select™cDNA subtraction kit (Clontech, Heidelberg, Germany) according to the manufacturer'sprotocol with a few modifications.

cDNA for PCR was prepared from tissues by RT-PCR using the primers 5'-ATGGGTCTCAGCCCCCACCT-3'and5' - CAAGTATTTCCCTCGTAGGA -3'aS forward and reverse primers, respectively, for IL-4 and 5'- CAAGAGACTTCCAGCCAGTTGC -3'AND 5'- TAGCCACTCCTTCTGTGACTCT-3' FOR IL-6 Using rat tissue cDNA as a template. The PCR amplification was performed using a PTC-100™(MJ, USA.). The PCR reaction used the following program: the reaction mixture was incubated at 95°C for 10 min, denatured for 1 min at 95°C, annealed for 30 sec at the optimal temperature (54°C for IL-4 and 49°C for IL-6) and extended at 72°C for 2 min. The program was repeated for 32 cycles and followed by an incubation at 72°C for 10 min for final extension. The results were analyzed by agarose gel electrophoresis.

### Histological analyses

Histological preparation of wound tissues was previously described by Drury and Wallington [46]. The rats were euthanized with an overdose of isoflurane, and tissue samples were collected from the wound sites to examine neutrophil infiltration into the wound area. Wounds were removed from four rats from each treatment group at 6 h, 24 h and at the end of the study period after wounding by cutting a square area that encompassed the entire wound site. The harvested tissues were immediately stored in a 10% formaldehyde solution in phosphate-buttered saline, washed in PBS, dehydrated in series of alcohol dilutions and embedded in paraffin. Microtome sections were cut vertically across the wound site and adhered to slides prior to staining with hematoxylin and eosin. Photographs of the sections in wound site were taken, and the images were digitized using Adobe Photoshop (Adobe Systems, Mountain View, CA). Neutrophil infiltration was scored according to Dommels et al. [47]. A rating score between 0 (no infiltration) and 10 (neutrophils involved in most areas) for inflammatory infiltrations, especially neutrophils, was assigned for each investigated section. Sections from at least five rats were carefully investigated for neutrophil infiltrations at the wound site.

### Statistical analysis

Statistical analysis was performed using the MINITAB software (MINITAB, State College, PA, Version 13.1, 2002). The data from experiments were first tested for normality using the Anderson Darling test and for variance homogeneity prior to any further statistical analysis. The data were normally distributed, and the variances were homogeneous; thus, one-way ANOVA was used to determine overall effects of the treatments followed by individual comparison using Tukey's Pairwise comparison. The results are expressed as the means ± standard deviations (SD). P-values > 0.05 were considered statistically non-significant, and *P-values *< 0.05 were considered statistically significant.

## Abbreviations

IL-1ß: Interleukin-1b; IL-6: Interleukin-6; GP: Glutathione; MDA: Malondialdehyde; NO: Nitric Oxide; ROS: Reactive Oxygen Species; PBMCs: Peripheral Blood Mononuclear Cells; TNF-α: Tumor Necrosis Factor alpha; WN: Wounded non-diabetic; WD: Wounded diabetic group; WDWP: Wounded diabetic group orally supplemented with the whey protein.

## Competing interests

The authors declare that they have no competing interests.

## Authors' contributions

**HE **designed the study, described histological changes, prepared figures, drafted the manuscript and performed the statistical analysis. **AS **was responsible of the animal model and histological investigations. **AAS **was responsible for PCR analysis. **AM **was responsible for the extraction and preparation of the WP. All authors read and approved the final manuscript.

## References

[B1] Ashcroft GS Horan MA Ferguson MW The effects of ageing on cutaneous wound healing in mammals J Anat 1995 187 1 26 PMC1167345 7591970

[B2] Singer AJ Clark RA Cutaneous wound healing N Engl J Med 1999 341 738 746 10.1056/NEJM199909023411006 10471461

[B3] Bradshaw AD Sage EH Lanza R, Langer R, Vacanti J Regulation of cell behavior by matricellular proteins Principles of Tissue Engineering 2000 San Diego: Acad

[B4] Altavilla D Saitta A Cucinotta D Galeano M Deodato B Colonna M Torre V Russo G Sardella A Urna G Campo GM Cavallar V Squadrito G Squadrito F Inhibition of lipid peroxidation restores impaired vascular endothelial growth factor expression and stimulates wound healing and angiogenesis in the genetically diabetic mouse Diabetes 2001 50 667 674 10.2337/diabetes.50.3.667 11246889

[B5] Clark R Cutaneous tissue repair. I. Basic biologic consideration J Am Acad Dermatol 1985 13 701 725 10.1016/s0190-9622(85)70213-7 2416789

[B6] Clark R Molecular and cellular biology of wound repair 1996 2 New York: Plenum Press 553 550

[B7] Graves DT Nooh N Gillen T Davey M Patel S Cottrell D Amar S IL-1 plays an important role in oral but not dermal, wound healing J Immunol 2001 167 5316 5320 10.4049/jimmunol.167.9.5316 11673547

[B8] Hall ED Yonkers PA Horan KL Correlation between attenuation of post-traumatic spinal cord ischemia and preservation of tissue vitamin E by the 21-aminosteroid U-Z4006F: Evidence for an in vivo antioxidant mechanism J Neurotrauma 1989 6 169 176 10.1089/neu.1989.6.169 2810381

[B9] Ashour M Salem S Hassaneen H EL-Gadban H Elwan N Awad A Basu TK Antioxidant status and insulin dependent diabetes mellitus (IDDM) J Clin Biochem Nutr 1999 26 99 107

[B10] Hsu WT Tsai LY Lin SK Hsiao K Chen BH Effects of diabetes duration and glycemic control on free radicals in children with type 1 diabetes mellitus Ann Clin Lab Sci 2006 36 174 178 16682514

[B11] Bounous G Whey protein concentrate (WPC) and glutathione modulation in cancer treatment Anti-Cancer Res 2000 20 4785 4792 11205219

[B12] Lands LC Grey VL Smountas AA The effect of supplementation with A cysteine donor on muscular performance J Appl Physiol 1999 87 1381 1385 10.1152/jappl.1999.87.4.1381 10517767

[B13] Ebaid H Badr G Metwalli A Immunoenhancing property of dietary un-denatured whey protein derived from three camel breeds in mice Biologia in press

[B14] David OL Breakthrough Technology Produces Concentrated Whey Protein with Bioactive Immunoglobulins Clin Nut Insights 1999 6 1 4

[B15] Ganong W Review of Medical Physiology 1997 18 Stamford, CT: Appleton & Lange

[B16] Ebaid H Hassnein K El-Feki M The un-denatured whey protein enhanced wound healing in mice J Egyp Germ Soc 2005 40 2 27

[B17] Badr G Ebaid H Mohany H Modulation of immune cell proliferation and chemotaxis towards CC chemokine ligands (CCL)-21 and CXC chemokine ligand (CXCL)-12 in un-denatured whey protein-treated mice J nutrit Biochem in press 10.1016/j.jnutbio.2011.11.006 22444498

[B18] Kindt T Goldsby R Osborne B Kuby Immunology 2007 6 WH Freeman and company. New York

[B19] Lim Y Levy MA Bray TM Dietary supplementation of N-acetylcysteine enhances early inflammatory responses during cutaneous wound healing in protein malnourished mice J Nutr Biochem 2006 17 328 336 10.1016/j.jnutbio.2005.08.004 16214328

[B20] Shen W Shi D Wand D Guo Y Hai S Yue Z Quinestrol Treatment Induced Testicular Damage via Oxidative Stress in Male Mongolian Gerbils (Meriones unguiculatus) Exp Anim 2011 60 445 453 10.1538/expanim.60.445 22041281

[B21] Niki E Yamamoto Y Komuro E Sato K Membrane damage due to lipid peroxidation Am J Clin Nutr 1991 53 201S 205S 10.1093/ajcn/53.1.201S 1985388

[B22] Marchetti J Metal complexes of bovine lactoferrin inhibit in Vitro replication of herpes simplex virus type 1 and 2 BioMetals 1994 11 89 94 10.1023/a:1009217709851 9542061

[B23] Kawasaki M Inhibition of Kappa-casein glycomacropeptide and lactoferrin of influenza virus hemagglutination Int J Cancer 1993 57 1214 1215 10.1271/bbb.57.1214 7763995

[B24] Wong CW Watson DL Immunomodulatory effects of dietary whey proteins in mice J Dairy Res 1995 62 359 368 10.1017/s0022029900031058 7601980

[B25] Bounous G Batist PG Immunoenhancing property of dietary whey protein In mice: Role Of glutathione Clin Invest Med 1989 12 154 161 2743633

[B26] Bickel M Nothen SM Freiburghaus K Shire D Chemokine expression in human oral keratinocyte cell lines and keratinized mucosa J Dent Res 1996 75 1827 1834 10.1177/00220345960750110301 9003228

[B27] Zhang X Kohli M Zhou Q Graves DT Amar S Short- and longterm effects of IL-1 and TNF antagonists on periodontal wound healing J Immuno 2004 173 3514 3523 10.4049/jimmunol.173.5.3514 15322216

[B28] Hubner G Brauchle M Smola H Madlener M Fassler R Werner S Differential regulation of pro-inflammatory cytokines during wound healing in normal and glucocorticoid-treated mice Cytokine 1996 8 548 556 10.1006/cyto.1996.0074 8891436

[B29] Nian M Lee P Khaper N Liu P Inflammatory cytokines and postmyocardial infarction remodeling Circ. Res 2004 94 1543 1553 10.1161/01.RES.0000130526.20854.fa 15217919

[B30] Park BK Lee S Seo JN Rhee JW Park JB Kim YS Choi IG Kim YE Lee Y Kwon HJ Protection of burn-induced skin injuries by the flavonoid kaempferol BMB Rep 2010 43 46 51 10.5483/bmbrep.2010.43.1.046 20132735

[B31] Rusu D Drouin R Pouliot Y Gauthier S Poubelle PE A bovine whey protein extract stimulates human neutrophils to generate bioactive IL-1Ra through a NF-kappaB- and MAPK-dependent mechanism J Nutr 2010 140 382 391 10.3945/jn.109.109645 20032479

[B32] Pradhan L Xuemei Cai X Wu S Nicholas D Andersen M Junaid M Patrick G Aristidis V Frank WL Gene Expression of Pro-Inflammatory Cytokines and Neuropeptides in Diabetic Wound Healing J Surg Res 2011 15 336 342 10.1016/j.jss.2009.09.012 PMC4376536 20070982

[B33] Silhi N Diabetes and wound healing J Wound Care 1988 7 47 51 10.12968/jowc.1998.7.1.47 9510751

[B34] Werner S Keratinocyte growth factor: A unique player in epithelial repair processes Cytokine Growth Factor Rev 1998 9 153 165 10.1016/s1359-6101(98)00010-0 9754709

[B35] Goodson WH Hunt TK Studies of wound healing in experimental diabetes J Surg Res 1977 22 221 227 10.1016/0022-4804(77)90137-8 14280

[B36] Ryan C Clark B Rosen A Patricia KV Hebda A Biochemical Markers Associated With Acute Vocal Fold Wound Healing: A Rabbit Model J Voice 2004 19 283 289 10.1016/j.jvoice.2004.04.003 15907442

[B37] Lawrence WT Physiology of the acute wound Clin Plast Surg 1998 25 321 340 9696896

[B38] Ehrlich HP Longaker MT Collagen considerations in scarring and regenerative repair Scarless Wound Healing 2000 New York, Marcel Dekker 99 113

[B39] McGrath MH Hundahl SA The spatial and temporal quantification of myofibroblasts Plast Reconstr Surg 1982 69 975 985 10.1097/00006534-198206000-00012 7043511

[B40] Lim Y Levy M Bray TM Dietary zinc alters early inflammatory responses during cutaneous wound healing in weanling CD-1 mice J Nutr 2004 134 811 816 10.1093/jn/134.4.811 15051830

[B41] Pari L Murugan P Tetrahydrocurcumin Prevents Brain Lipid Peroxidation in Streptozotocin-Induced Diabetic Rats J Med Food 2007 10 323 329 10.1089/jmf.2006.058 17651069

[B42] Schwentker A Yoram V Richard W Timothy R Billiar Nitric oxide and wound repair: role of cytokines? Nitric Oxide 2002 7 1 10 10.1016/s1089-8603(02)00002-2 12175813

[B43] Clark KD Lu Z Strand MR Regulation of melanization by glutathione in the moth Pseudoplusia includens Insect Biochem Molecul Biol 2010 40 460 467 10.1016/j.ibmb.2010.04.005 20417279

[B44] Oliveira DM Silva-Teixeira DN Araujo-Filho R Goes AM Antigenic stimulation is more efficient than LPS in inducing nitric oxide production by human mononuclear cells on the in vitro granuloma reaction in schistosomiasis Braz J Med Biol Res 1999 32 1437 45 10.1590/s0100-879x1999001100015 10559846

[B45] Okhawa H Ohishi N Yagi K Assay for lipid peroxides in animal tissues by thiobarbituric acid reaction Anal Biochem 1979 95 351 358 10.1016/0003-2697(79)90738-3 36810

[B46] Drury RAB Wallington EA Carleton's histological technique 1980 5 Oxford, Oxford University Press 188 189

[B47] Dommels YE Butts CA Zhu S Davy M Martell S Hedderley D Barnett MP McNabb WC Roy NC Characterization of intestinal inflammation and identification of related gene expression changes in mdr1a^(-/-) ^mice Genes Nutr 2007 2 209 223 10.1007/s12263-007-0051-4 PMC2474946 18850176

